# Pulsed field ablation and cryoballoon ablation for pulmonary vein isolation: insights on efficacy, safety and cardiac function

**DOI:** 10.1007/s10840-024-01748-4

**Published:** 2024-01-26

**Authors:** Manuel Rattka, Evangelos Mavrakis, Dimitra Vlachopoulou, Isabel Rudolph, Christina Kohn, Jan Bohnen, Loubna Yahsaly, Johannes Siebermair, Reza Wakili, Christiane Jungen, Tienush Rassaf, Shibu Mathew

**Affiliations:** 1 Department of Cardiology and Vascular Medicine, West German Heart and Vascular Center Essen, University of Duisburg-Essen, Hufelandstraße 55, 45147 Essen, Germany; 2 Krankenhaus Goettlicher Heiland, Vienna, Austria; 3 Department of Cardiology and Vascular Medicine, University Hospital Frankfurt, Goethe-University Frankfurt, Frankfurt am Main, Germany

**Keywords:** Atrial fibrillation, Ablation, Pulsed field ablation, Cryoballoon ablation; LVEF

## Abstract

**Background:**

Pulmonary vein isolation (PVI) has become the cornerstone treatment of atrial fibrillation (AF). While in cryoablation cell damage is caused by thermal effects, lately, pulsed field ablation (PFA) has been established as a novel non-thermal tissue-specific ablation modality for PVI. However, data comparing outcomes of patients undergoing either PFA or cryoballoon ablation (CBA) for primary PVI are sparse.

**Methods:**

Consecutive patients with AF undergoing PVI by either CBA or PFA were included in the analysis. The primary outcome was the time to AF/AT recurrence. For secondary outcomes, clinical and periprocedural parameters were compared.

**Results:**

In total, outcomes of 141 AF patients treated by PFA (94 patients) or CBA (47 patients) were compared. After 365 days, 70% of patients in the PFA group and 61% of patients in the CBA group were free from AF/AT (*HR* 1.35, 95% *CI* 0.60–3.00; *p* = 0.470). No deaths occurred. While symptoms alleviated in both groups, only after PFA, we observed significant improvement of left atrial volume index (PFA group baseline: 40 [31;62] ml/m^2^, PFA group follow-up: 35 [29;49] ml/m^2^; *p* = 0.015), NT-pro BNP levels (PFA group baseline: 1106 ± 2479 pg/ml, PFA group follow-up: 1033 ± 1742 pg/ml; *p* = 0.048), and left ventricular ejection fraction (LVEF) (PFA group baseline: 55 [48;60] %, PFA group follow-up: 58 [54;63] %; *p* = 0.006). PVI by PFA was the only independent predictor of LVEF improvement.

**Conclusion:**

In our study, we show that CBA and PFA for PVI are of similar efficacy when it comes to AF recurrence. However, our findings suggest that PFA rather than CBA might induce left atrial reverse remodeling thereby contributing to left ventricular systolic function.

## Introduction

Pulmonary vein isolation (PVI) for atrial fibrillation (AF) is a hallmark of AF treatment and can be achieved by a variety of methodical approaches. The efficacy of conventional ablation techniques improved over the past years, but freedom from atrial arrhythmia is still between 71 and 86% in patients with paroxysmal AF after 1 year [1–3]. Therefore, alternative ablation techniques are needed to improve long-term ablation success. While thermal ablation modalities, such as cryoballoon ablation (CBA) or radiofrequency ablation (RFA), have been established as cornerstone therapy over the past years, lately, pulsed field ablation (PFA) has been introduced as a novel non-thermal tissue-specific ablation modality for PVI. By application of ultra-rapid electrical pulses, PFA generates an electrical field disrupting cell membranes by creating nanoscale pores, ultimately leading to cell death. Remarkably, at a defined energy threshold, electroporation has been shown to be nearly myocardium-specific, thereby minimizing the potential collateral damage to non-target tissue, while allowing effective ablation of cardiomyocytes in a defined area [4]. However, data comparing periprocedural and rhythm outcomes of patients undergoing pulmonary vein isolation for atrial fibrillation by either cryoballoon ablation or pulsed field ablation are limited. Additionally, information regarding the effect of PFA for PVI on atrial and ventricular structure and function are sparse, especially in comparison to CBA.

## Methods

### Study population

We conducted a case-control study taking into account patients treated at the Department of Cardiology and Angiology at Essen University Medical Center, Essen, Germany, with paroxysmal or persistent atrial fibrillation with an indication for first-do PVI according to the current ESC guideline [5]. Consecutive patients undergoing either cryoballoon ablation (POLARx™, Boston Scientific, Massachusetts, USA) or pulsed field ablation (FARAWAVE™, Boston Scientific, Massachusetts, USA), were included. The ablation modality was chosen at the discretion of the interventionalist. Patients with decompensated heart failure, cardiogenic shock, or patients scheduled for repeat ablation were not eligible. The study complies with the Declaration of Helsinki and was approved by the local ethics committee (University of Duisburg-Essen).

### Preprocedural management and ablation procedure

Preprocedural management was performed as previously described [6, 7]. In brief, left atrial thrombus was ruled out by transesophageal echocardiography before the procedure in all patients prior to PVI. Otherwise, no preprocedural imaging was routinely acquired. Vitamin K antagonists were administered uninterruptedly with a target INR of 2.0–2.5 at the time of procedure. Patients treated with non-VKA oral anticoagulants were advised to hold their anticoagulant <24 h prior to the ablation procedure. All procedures were performed under deep sedation with continuous heparin administration during the procedure by activated clotting time goal of 300-350ms. In all patients a diagnostic catheter was placed in the coronary sinus, and an additional quadripolar catheter in the right ventricle, respectively for phrenic nerve capture if required. The respective ablation catheter was advanced to the left atrium after a single transseptal puncture. After successful transseptal puncture and introduction of the respective steerable sheath into the left atrium pulmonary vein (PV), angiography was performed to capture PV anatomy. In the PFA group, a 12-French over-the-wire PFA ablation catheter (FARAWAVE™, Boston Scientific, Massachusetts, USA) was used in either a flower or basket configuration delivering the energy in a set of microsecond-scale biphasic pulses of 1800–2000 V in bipolar approach across all electrodes. Each application was made of five pulse packets delivered over a few seconds. Applications were repeated eight times per vein, four times in flower, four times in basket configuration, with repositioning and/or rotation of the catheter every two applications. Before energy delivery the ablation catheter was positioned at the proximal border of the PV ostium according to the PV angiography to ensure circumferential PV ostial and antral coverage as good as possible. The procedural endpoint of complete PVI was confirmed by bidirectional conduction block after PFA applications. In patients undergoing cryoballoon ablation, PVI was performed using a 28-mm cryoballoon catheter (POLARx™, Boston Scientific, Massachusetts, USA) under fluoroscopic guidance. To achieve PV occlusion, the CB was inflated proximal to the PV ostium and complete occlusion of the PV ostium was verified by injections of contrast media. The standard freeze-cycle duration was 180 s. During PVI of the septal PVs, continuous phrenic nerve (PN) pacing was performed using a diagnostic catheter placed within the superior vena cava. Pacing was set at maximum output and pulse width. PN capture was also monitored by the tactile feedback of diaphragmatic contraction. After PVI, entrance and exit block were confirmed by either placing the PolarMap^TM^ catheter or the FARAWAVE^TM^ catheter within the PVs and pacing maneuvers were performed as recommended. Additionally, pre- and post-ablation three-dimensional ultra-high-density mapping was conducted at the discretion of the interventionalist in both groups.

### Postprocedural management and clinical follow-up

Echocardiography was performed in every patient immediately after the procedure and before hospital discharge to rule out pericardial effusion or pericardial tamponade. All patients underwent a neurological examination directly after the procedure to detect potential focal neurological deficits. Oral anticoagulation was resumed in the evening after the intervention in the absence of groin complications. Anti-arrhythmic drugs were discontinued immediately after the procedure. At the first day after the procedure a 12-lead ECG was written to determine the current rhythm. Patients were scheduled for outpatient clinic visits including clinical assessment, transthoracic echocardiography, and 7-day Holter monitoring at 3 and 6 months after the procedure and thereafter every 6 months. Any documented sustained atrial tachyarrhythmia on 12-lead ECG or any tachyarrhythmia of 30 s or longer on Holter ECG was counted as AF recurrence. All patients were off anti-arrhythmic drugs. A blanking period of 90 days was applied.

### Primary and secondary outcomes

The primary outcome was the time to AF/AT recurrence. Secondary outcomes were procedural parameters and periprocedural complications. Furthermore, clinical parameters at baseline and at the end of follow-up were compared between both groups. Start of the follow-up period was defined as the day of the ablation procedure.

### Statistical analysis

The significance of differences of numeric values was calculated by *t* test if normal distribution with equal variance was given. Numeric variables that were not normally distributed were analyzed by Mann–Whitney rank sum test and described as median and first to third interquartile range. Categorical variables were described as absolute and relative values and analyzed by *χ*^2^ test or Fisher’s exact test, as appropriate. Kaplan–Meier analysis was used to assess the time to event and groups were compared using the Cox proportional hazard model. For identification of independent predictors of LVEF improvement, as defined by any numeric LVEF improvement in the echocardiographic examination at the end of follow-up, baseline characteristics were analyzed by univariate logistic regression. Parameters with a *p* ≤ 0.1 were further tested for independency by multivariate logistic regression. A *p* < .05 was considered to be statistically significant. Statistical assessment was performed by SPSS Statistics 27 software (Version 2020; IBM).

## Results

### Study population

A total of 141 consecutive patients, 47 patients treated by CBA and 94 by PFA, were included in our study. Patients had a median age of 63 years and were predominantly male (66%). Type of atrial fibrillation (AF) was equally distributed between ablation groups (*p* = 0.575). Patients treated by PFA were significantly more often diagnosed with arterial hypertension (CBA group: 26 out of 47 patients, PFA group: 73 out of 94 patients; *p* = 0.006), while diabetes mellitus was observed more often in the CBA group (CBA group: 15 out of 47 patients, PFA group: 16 out of 94 patients; *p* = 0.044). Otherwise, there were no significant differences in baseline characteristics between both groups as listed in Table 1.

**Table 1:** Demographic and baseline characteristics of patients undergoing either pulsed field- or cryoballoon ablation for atrial fibrillation

	PFA group	CBA group	*p*-value
Number of patients	94	47	
Age, years	63 ± 12	64 ± 12	0.478
Female sex, *n* (%)	36 (38)	12 (26)	0.132
Hypertension, *n* (%)	73 (78)	26 (55)	0.006
BMI > 25 kg/m^2^	41 (44)	27 (57)	0.121
Dyslipidemia, *n* (%)	59 (63)	32 (68)	0.534
Diabetes mellitus, *n* (%)	16 (17)	15 (32)	0.044
Prior MI, *n* (%)	6 (6)	5 (11)	0.374
TIA/stroke, *n* (%)	7 (7)	2 (4)	0.465
Vascular disease, *n* (%)	33 (35)	21 (45)	0.270
AADs, *n* (%)			
• none	66 (70)	31 (66)	0.667
• class I	22 (24)	11 (23)	
• class III	6 (6)	5 (11)	
Oral anticoagulation, *n* (%)	94 (100)	47 (100)	1.000
Type of AF, *n* (%)			
• paroxysmal	53 (56)	24 (51)	0.575
• persistent	28 (30)	18 (38)	
• long-standing persistent	13 (14)	5 (11)	
CHA_2_DS_2_-VASc Score	3 [1; 4]	3 [1; 4]	0.438
HAS-BLED Score	1 [0; 2]	2 [1; 2]	0.173

Mean ± standard deviation; median [interquartile range]; number (percent)

*PFA* pulsed field ablation, *CBA* cryoballoon ablation, *BMI* body mass index, *MI* myocardial infarction, *TIA* transitory ischemic attack, *AAD* anti-arrhythmic drug, AF atrial fibrillation

### Arrhythmia recurrence

**Fig. 1:**
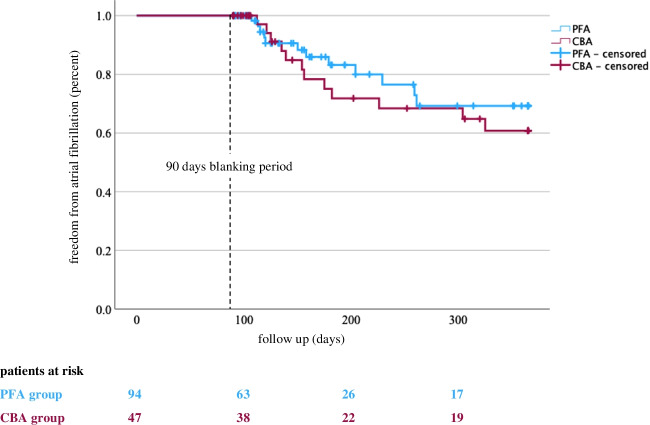
Comparison of arrhythmia recurrence between AF patients undergoing either CBA or PFA for PVI

Arrhythmia recurrence was compared between AF patients undergoing either CBA or PFA for PVI. There was no significant difference after 365 days between the groups (*HR* 1.35, 95% *CI* 0.60–3.00; *p* = 0.470; Fig. 1). In total, after PFA, 70% had no atrial fibrillation/atrial tachycardia (AF/AT) recurrence after 365 days compared to 61% after CBA. For patients with paroxysmal AF, 87% of patients from the PFA group and 83% of patients from the CBA group; and for persistent AF, 57% of patients from the PFA group and 58% of patients from the CBA group were still free from AF. With regard to long-standing persistent AF, 27% of patients from the PFA group and 20% of patients from the CBA group had no AF/AT recurrence. No deaths occurred in both groups.

### Procedural parameters and periprocedural complications

Assessment of procedural parameters showed no significant difference for total procedure time and fluoroscopic time between both groups. However, the amount of contrast dye used during the procedure (CBA group, 154 ± 82 ml*; PFA group,* 99 ± 66 ml*; p* < 0.001) was significantly lower in the PFA group. There were no significant differences in periprocedural complications (CBA group, 1 patient*; PFA group,* 4 patients*; p* = 0.664). All patients had full recovery. Procedural parameters are detailed in Table 2.

**Table 2:** Procedural parameters and periprocedural complications

	PFA group	CBA group	*p*-value
Isolated veins	376 (100)	188 (100)	1.000
Contrast dye (ml)	99 ± 66	154 ± 82	<0.001
Fluoroscopy time (min)	26 ± 9	23 ± 9	0.059
Total procedure time (min)	162 ± 64	163 ± 62	0.931
Complications, *n* (%)	4 (4)	1 (2)	0.664
TIA/stroke	2 (2)	0 (0)	
Transient PNP	0 (0)	1 (2)	
Pericardial tamponade	1 (1)	0 (0)	
Air embolism	1 (1)	0 (0)	

Mean ± standard deviation; number (percent)

*PFA* pulsed field ablation, *CBA* cryoballoon ablation, *MI* myocardial infarction, *TIA* transitory ischemic attack, *PNP* phrenic nerve palsy

### Clinical parameters at baseline and at the end of follow-up

To further evaluate the effect of both ablation modalities on clinical outcome, AF patients’ symptoms as measured by NYHA- and EHRA-class, as well as serum NT-pro BNP levels, left ventricular ejection fraction (LVEF), and left atrial volume index (LAVI), were assessed at baseline and at the end of follow-up.

Median EHRA-class (CBA group baseline (CBA-bl): 3 [2; 3], CBA group follow-up (CBL-fu): 0 [0; 2]; *p* < 0.001; PFA group baseline (PFA-bl): 3 [2; 3], PFA group follow-up (PFA-fu): 1 [0;2]; *p* < 0.001) and median NYHA-class (CBA-bl: 2 [2; 3], CBA-fu: 2 [1; 2]; *p* = 0.002; PFA-bl: 2 [2; 3], PFA-fu: 1 [1; 2]; *p* < 0.001) improved significantly in both groups. Remarkably, we observed a significant decrease of NT-pro BNP serum levels (PFA-bl: 1106 ± 2479 pg/ml, PFA-fu: 1033 ± 1742 pg/ml; *p* = 0.048) and left atrial volume index (PFA-bl: 40 [31; 62] ml/m^2^, PFA-fu: 35 [29; 49] ml/m^2^; *p* = 0.015) in patients treated with PFA. LVEF increased in this population (PFA-bl: 55 [48; 60] %, PFA-fu: 58 [54; 63] %; *p* = 0.006). This was only seen in patients undergoing PVI by PFA, while there were no significant differences in patients treated by CBA.

There were no significant differences between the groups both at baseline and at the end of follow-up. Clinical parameters are shown in Table 3.

**Table 3:** Clinical parameters at baseline and at the end of follow-up

	PFA group	CBA group	*p*-value
NYHA class			
• Baseline	2 [2; 3]	2 [2; 3]	0.532
• Follow-up	1 [1; 2]***	2 [1; 2]**	0.226
EHRA class			
• Baseline	3 [2; 3]	3 [2; 3]	0.473
• Follow-up	1 [0; 2]***	0 [0; 2]***	0.374
NT-pro BNP (pg/ml)			
• Baseline	1106 ± 2479	1137 ± 1358	0.935
• Follow-up	1033 ± 1742*	1178 ± 1314	0.713
LVEF (%)			
• Baseline	55 [48; 60]	55 [54; 59]	0.840
• Follow-up	58 [54; 63]**	55 [53; 59]	0.323
LAVI (ml/m^2^)			
• Baseline	40 [31; 62]	40 [26; 57]	0.330
• Follow-Up	35 [29; 49]*	36 [26; 40]	0.884

*PFA* pulsed field ablation, *CBA* cryoballoon ablation, *NYHA* New York Heart Association, *EHRA* European Heart Rhythm Association, *BNP* brain natriuretic peptide, *LVEF* left ventricular ejection fraction, *LAVI* left atrial volume index

Mean ± standard deviation; median [interquartile range]

* *p* < 0.05; ** *p* < 0.01; *** *p* < 0.001

### Identification of predictors of left ventricular ejection fraction improvement

To identify independent predictors of LVEF improvement, we performed uni- as well as multivariate logistic regression analysis. In univariate logistic regression analysis, we identified sex and PVI by PFA as potential predictors of LVEF improvement. However, in the multivariate analysis only PVI by PFA remained independently and significantly associated with improvement of LVEF (*HR* 3.70, 95% *CI* 1.19–11.49; *p* = 0.024; Table 4).

**Table 4:** Identification of predictors of LVEF improvement

Variables	Univariate analysis			Multivariate analysis		
	*HR*	95% *CI*	*p*-value	*HR*	95% *CI*	*p*-value
Sex (female)	0.21	0.05–0.83	0.026	0.38	0.12–1.17	0.092
Age (years)	1.05	0.98–1.11	0.145			
Type of AF	0.99	0.33–2.92	0.982			
BMI > 25 kg/m2	1.02	0.22–4.72	0.979			
Hypertension	1.86	0.31–11.31	0.499			
DM	0.92	0.19–4.52	0.916			
Dyslipidemia	0.39	0.09–1.65	0.200			
CAD	0.54	0.13–2.23	0.396			
Prior MI	0.22	0.02–2.63	0.233			
SR at baseline	1.07	0.24–4.76	0.932			
PVI by PFA	3.53	0.81–15.38	0.093	3.70	1.19–11.49	0.024

*LVEF* left ventricular ejection fraction, *HR* hazard ratio, *CI* confidence interval, *AF* atrial fibrillation, *BMI* body mass index, *DM* diabetes mellitus, *CAD* coronary artery disease, *MI* myocardial infarction, *SR* sinus rhythm, *PVI* pulmonary vein isolation, *PFA* pulsed field ablation

## Discussion

In this study, we compared outcomes of patients undergoing either cryoballoon ablation (CBA) or pulsed field ablation (PFA) for pulmonary vein isolation (PVI) in atrial fibrillation (AF) patients.

We found that after 365 days, there was no significant difference in AF/AT recurrence between both groups. The 1-year success rates between both groups were similar for patients with paroxysmal AF (PFA-group: 87%; CBA-group: 83%), persistent AF (PFA-group: 57%; CBA-group: 58%), and long-standing persistent AF (PFA-group: 27%; CBA-group: 20%). Additionally, there was no significant difference in periprocedural complications. The amount of contrast dye used was lower in PFA patients. There was no difference in total procedure time. Compared to baseline, AF patients undergoing PFA showed decreased NT-pro BNP levels, a lower left atrial volume index, and increased left ventricular ejection fraction at the end of follow-up, whereas there were no differences for CBA patients.

### Rhythm outcome

PFA has been introduced as a novel non-thermal and tissue-specific ablation modality achieving PVI by application of rapidly alternating high electrical fields to atrial tissue, thereby inducing nanopores followed by cell death [4]. Current data from the MANIFEST-PF registry and the PULSED AF Pivotal trial showed comparable efficacy rates after 12 months as compared to thermal ablation approaches [8–10]. While 78% of patients included in the MANIFEST-PF registry were free from atrial arrhythmia after 365 days, in the PULSED AF Pivotal trial, 66% of patients with paroxysmal AF and 55% of patients with persistent AF were free from any atrial arrhythmia after 1 year [8, 9]. Urbanek et al. were the first to show that single-shot ablation for PVI by PFA is of similar efficacy and safety as compared to CBA. They found that after 1-year, approximately 83% of patients with paroxysmal AF who underwent PFA and 80% of patients who underwent CBA were still free from AF. For patients with persistent AF 1-year success rates were 67% for PFA and 71% for CBA, respectively [11]. The ADVENT-trial, which compared PFA with conventional thermal ablation in paroxysmal AF patients only, reported a 1-year treatment success in 73% of patients treated by PFA and in 71% after thermal ablation [12]. Schipper et al. describe freedom from atrial arrhythmia in 74% of patients who received PFA and in 72% of patients treated by CBA after 365 days [13]. In our study, we observed freedom from AF in 70% of patients in the PFA group and 61% of patients in the CBA group at the end of follow-up. If analyzed by AF type, 87% of paroxysmal AF patients treated by PFA and 83% of patients who underwent CBA were still free from any atrial arrhythmia after 365 days, while freedom from AF in patients with persistent AF was 57% in the PFA group and 58% in the CBA group. This was even lower in patients with long-standing persistent AF (PFA-group: 27%; CBA-group: 20%). Consequently, overall our data are in line with the previously mentioned studies and substantiate that CBA and PFA for PVI appear to be of similar efficacy in the treatment of AF [8–11]. Additionally, we observed that AF/AT recurrence after PVI is less likely in patients with paroxysmal AF than in patients with persistent and long-standing persistent AF. This underscores that early rhythm control is crucial for long-term maintenance of sinus rhythm [14].

### Procedural parameters

An analysis of procedural parameters showed that the amount of contrast dye used was significantly lower in the PFA group, whereas we did not observe differences regarding the total procedure time. In comparison to the study of Urbanek et al., the total procedure time was longer in our patient population [11]. Supposedly, this is attributable to the use of 3-dimensional ultra-high-density mapping before and after the ablation procedure, which was conducted at the discretion of the interventionalist. That might also have leveled a potential difference in procedure time between both groups. Furthermore, we observed that PFA was associated with a lower amount of contrast dyes used, which is not surprising since PFA does not rely on confirming pulmonary vein occlusion before ablation by injection of contrast dye. Consequently, PFA for PVI might be more suitable for patients with known chronic kidney injury compared to CBA since the exposition to iodinated contrast dye is lower [15]. As for periprocedural complications, our data are comparable with the adverse event rates reported by Schipper et al. and Urbanek et al. [11, 13].

### Clinical outcomes

To evaluate the impact of PFA and CBA for PVI in AF patients on clinical outcomes, symptoms related to arrhythmia as measured by EHRA-class and symptoms related to heart failure as measured by NYHA-class were assessed at baseline and at the end of follow-up. Additionally, NT-pro BNP levels, left atrial volume index (LAVI), and left ventricular ejection fraction (LVEF) were compared to detect potential effects on cardiac function.

We found that symptoms as measured by NYHA-class and EHRA-class ameliorated significantly after PVI at the end of follow-up, which has previously been shown in other studies [16, 17]. This was the same for AF patients who underwent either PFA or CBA for PVI. There was no significant difference between both groups.

Furthermore, the analysis of LAVI showed that at the end of follow-up in the PFA group there were signs of left atrial structural reverse-remodeling, while there was no difference in the CBA group. It is known that atrial remodeling is pivotal for the occurrence and development of AF, and it has been shown that PVI for AF can induce reverse-remodeling, especially in paroxysmal AF. For instance, Wang et al. demonstrated that both radiofrequency ablation and CBA are associated with atrial reverse-remodeling and that, interestingly, CBA even might outperform RFA in this context [18]. Our results suggest that PFA might even be superior to CBA regarding left atrial reverse remodeling. This might be related to the tissue-specificity of PFA consequently reducing the *collateral damage* inflicted on the left atrium, as compared to CBA [19].

Remarkably, comparison of LVEF revealed that left ventricular systolic function improved in the PFA group but not in the CBA group. Previous studies have shown that atrial reverse-remodeling and improvement of LVEF after restoration of sinus rhythm can be expected to occur after successful PVI for AF, even in patients with normal LVEF [20]. However, the mechanism is only poorly understood. One possible explanation is that left atrial reverse-remodeling improves left ventricular filling [20–22]. Since multivariate regression analysis identified PFA ablation as the only variable significantly and independently associated with LVEF improvement, we hypothesize that left atrial reverse-remodeling improves left ventricular filling thereby contributing to left ventricular systolic function. The finding of left atrial reverse-remodeling and the idea of improved cardiac function is substantiated by the significant decrease of NT-pro BNP levels at the end of follow-up only in the PFA group. However, further studies are warranted to verify this hypothesis.

## Limitations

As this is a single center case control study, comparing the effects of CBA and PFA for PVI in AF patients on AF-freedom and periprocedural efficacy and safety, it inherently has limitations. Due to the novelty of the PFA system, operators had no previous experience using this technology, which might affect the procedural parameters and consequently, a learning curve must be considered. Moreover, since this is a study from a single center, only a limited number of patients could be included. Consequently, our results must be interpreted as hypothesis generating. Multicenter studies are of the essence to verify our results.

## Conclusion

In this study, we compared cryoballoon ablation (CBA) and pulsed field ablation (PFA) for pulmonary vein isolation (PVI) in atrial fibrillation (AF) regarding rhythm outcomes, procedural parameters, and functional and clinical outcomes. While CBA and PFA for PVI are of similar efficacy when it comes to AF recurrence, PFA was associated with a lower amount of contrast dye used during the intervention. Additionally, our results indicate that PFA rather than CBA might induce left atrial reverse remodeling thereby contributing to left ventricular systolic function.
